# TNFR1 Signaling Contributes to T Cell Anergy During *Staphylococcus aureus* Sepsis

**DOI:** 10.3389/fcimb.2018.00259

**Published:** 2018-08-03

**Authors:** Camila Ledo, Cintia D. Gonzalez, Carolina V. Poncini, Marta Mollerach, Marisa I. Gómez

**Affiliations:** ^1^Instituto de Investigaciones en Microbiología y Parasitología Médica, Consejo Nacional de Investigaciones Científicas y Tecnológicas, Universidad de Buenos Aires, Buenos Aires, Argentina; ^2^Departamento de Microbiología, Parasitología e Inmunología, Facultad de Medicina, Universidad de Buenos Aires, Buenos Aires, Argentina; ^3^Departamento de Investigaciones Biomédicas y Biotecnológicas, Centro de Estudios Biomédicos, Biotecnológicos, Ambientales y de Diagnóstico, Universidad Maimónides, Buenos Aires, Argentina; ^4^Cátedra de Microbiología, Facultad de Farmacia y Bioquímica, Universidad de Buenos Aires, Buenos Aires, Argentina; ^5^CONICET, Buenos Aires, Argentina

**Keywords:** TNFR1, *Staphylococcus aureus*, sepsis, MDSC, anergy, T cell

## Abstract

Early research on sepsis has focused on the initial hyper-inflammatory, cytokine mediated phase of the disorder whereas the events that govern the concomitant and subsequent anti-inflammatory compensatory response are not completely understood. In this context, the putative participation of TNFR1-mediated signaling in the immunosuppressive phase of *Staphylococcus aureus* sepsis has not been elucidated. The aim of this study was to determine the role of TNFR1 in directing the immune dysfunction during *S. aureus* sepsis and the potential contribution of MDSC to this process. Using a model of sepsis of peritoneal origin and *tnfr*1^−/−^ mice, we demonstrated that during staphylococcal sepsis CD4^+^ T cell anergy is significantly dependent on TNFR1 expression and that signaling through this receptor has an impact on bacterial clearance in the spleen. MDSC played a major role in the generation of anergic CD4^+^ T cells and their accumulation in the spleen during *S. aureus* sepsis correlated with IL-6 induction. Although TNFR1 signaling was not required for MDSC accumulation and expansion in the spleen, it determined the *in vivo* expression of Arginase 1 and iNOS, enzymes known to participate in the suppressive function of this population. Moreover, our data indicate that TNFR1-mediated IL-10 production may modulate MDSC function during staphylococcal sepsis. Taken together these results indicate that TNFR1 plays a critical role on T cell dysfunction during *S. aureus* sepsis by regulating immunomodulatory mediators in MDSC. The role of TNFR1-mediated signaling during the immunosuppressive phase of staphylococcal sepsis should be considered when designing novel alternative therapeutic approaches.

## Introduction

Sepsis is a major public health issue and despite improved patient management and supportive care it remains as the leading cause of death in the intensive care units with an estimation of 15–30 million cases worldwide every year (Adhikari et al., 2010; Mayr et al., 2014; Delano and Ward, 2016; Fleischmann et al., 2016) and an incidence of 450 per 100,000 inhabitants (Fleischmann et al., 2016) with nearly 20% mortality rate. The etiology of sepsis has been changing over the last decades with a raising incidence of the gram positive cocci *Staphylococcus aureus* (Sampedro and Bubeck Wardenburg, 2017). The origin of sepsis can be related to hospital interventions as well as to complications of community-acquired infections that become invasive. In this context, the high incidence of methicillin resistant isolates, from hospital and community environments, have added complexity to the situation.

Early research on sepsis has focused on the initial hyper-inflammatory, cytokine mediated phase of the disorder (Opal et al., 1997; Abraham et al., 1998; van Dissel et al., 1998). More recent findings indicate that, although in the beginning of sepsis the balance favors an inflammatory profile (Powers and Bubeck Wardenburg, 2014), anti-inflammatory responses are initiated during the hyper-inflammatory phase leading to subsequent immunosuppression (Tang et al., 2010; Boomer et al., 2014). These varying states of immune paralysis are characterized by impaired immune surveillance and the development of persistent, recurrent, secondary, and nosocomial infections which facilitate protracted events that often lead to death in patients that have survived the initial period of sepsis (Monneret et al., 2011; Otto et al., 2011).

The events that govern immunosuppression during sepsis are not completely elucidated. Moreover, the role that TNF-α signaling might have in modulating the anti-inflammatory response has not been determined. Myeloid derived suppressor cells (MDSC) have been described as an immature population of cells that is able to suppress T cell responses in polymicrobial sepsis and during chronic and persistent *S. aureus* infections (Heim et al., 2014; Tebartz et al., 2015) and it has been described that TNF-α participates in their accumulation and activation during chronic inflammation (Sade-Feldman et al., 2013). We have previously shown the ability of *S. aureus* to modulate TNF-α/TNFR1 signaling during local and systemic infections (Gómez et al., 2004; Giai et al., 2013). Therefore, we hypothesize that TNFR1 signaling could have a role in directing the immunosuppression during *S. aureus* sepsis and that MDSC may participate in that response.

## Materials and methods

### Bacterial strains and culture conditions

*S. aureus* strain FPR3757 (pulsotype USA300, ST8, SCCmec type IV-spa type 008, PVL positive) was provided by Dr. Alice Prince (Columbia University, NY, USA). *S. aureus* strain Sa30 (ST30, SCCmec type IVc-spa type 019, PVL positive) was previously described (Fernandez et al., 2013). Bacteria were grown on tryptone soy agar (TSA) or tryptone soy broth (TSB). Bacteria were grown at 37°C with agitation until an OD_600_ of 0.8: washed and suspended in phosphate buffer (PBS).

### Animals and housing

Mice were obtained from and maintained in the animal facility of the Department of Microbiology, School of Medicine, University of Buenos Aires. All the procedures involving laboratory animals were approved by the Institutional Committee for Use and Care of Laboratory Animal (CICUAL) of the School of Medicine, University of Buenos Aires (Approval numbers 2737/14 and 2865/15) and followed internationally accepted guidelines (National Institutes of Health, 1996). Animals were maintained in a conventional facility, with controlled temperature (22 ± 1°C), controlled humidity (55%), a 12:12 h light/dark cycle and they were fed *ad libitum*. Procedures were performed in an experimental room within the mouse facility. Mice were euthanized using CO_2_. The number of mice required for each experiment was determined based on preliminary experiments and the desired statistical significance. Cumulative data from 2 to 4 independent experiments with small randomly chosen groups (control and experimental groups) of 3 to 6 animals are shown. The weight of the mice used was in accordance with their age. They showed good mobility and no differences in behavior were observed after manipulations. In this work molecular markers were evaluated in live animals and postmortem. The levels of pro-inflammatory cytokines in plasma from naïve mice were in the range of expected basal levels (IL-6: 0–50 pg/ml; TNF-α: 0–100 pg/ml).

### Mouse model

C57BL/6 and TNFR1 deficient (*tnfr*1^−/−^) mice (6 weeks old, 18-20 grams) were intraperitoneally inoculated with 200 μl of *S. aureus* containing 4 × 10^7^ CFU. The control groups were inoculated with PBS. Blood samples were obtained by puncture of the mandibular vein at different times before and after inoculation. Plasma was stored at −80°C for subsequent IL-10 and IL-6 quantification. The spleen was removed from mice under sterile conditions and single-cell suspensions were prepared by homogenization through a sterile stainless steel mesh. To assess the bacterial load, aliquots of the infected spleen were serial diluted and cultured on TSA plates.

### *In vivo* depletion of MDSC

Mice received a single intraperitoneal injection of 5′Fluorouracil (5FU, 50 mg/kg), a dose proven to specifically deplete MDSC without affecting other splenic cell populations (Vincent et al., 2010; Poncini and González-Cappa, 2017), at day 4 post-inoculation with *S. aureus*. MDSC depletion was confirmed by flow cytometry analysis after staining spleen cells with antibodies against CD11b, Gr-1, Ly6C, and Ly6G. Mice treated with 5FU evidenced Monocytic/Granulocytic MDSC proportions equivalent to non-infected mice.

### Flow cytometry

Single-cell suspensions were incubated with fluorescently-labeled mAbs for 30 min at 4°C. The following mAbs were used: phycoerythrin-labeled anti-CD4, fluorescein isothiocyanate-labeled anti-CD3 (Ligatis), allophycocyanin-labeled anti-CD8, Alexa 488-labeled anti-Gr1, phycoerythrin-cyanin 7-labeled anti-Gr1, phycoerythrin-labeled anti-CD11b, allophycocyanin-labeled anti-Ly6G and phycoerythrin-cyanin 7-labeled anti-Ly6G (Biolegend). Cells were washed and fixed in 1% paraformaldehyde. For intracellular staining, cells were cultured in the presence of monensin (2 μM; Biolegend) for 5 h. After surface staining, cells were incubated with phycoerythrin-labeled anti-CD11b and phycoerythrin-cyanin 7-labeled anti-Gr1 for 30 min at 4°C. Then, the cells were fixed (4% PFA) and permeabilized (PBS, 0.5% saponin, 10% FBS). The Abs used were anti-Arginase, anti-iNOS (Santa Cruz Biotechnology), and Alexa488-labeled anti-rabbit IgG-Alexa (Invitrogen). For Foxp3 detection, cells were stained using the T regulatory (Treg) cell detection kit (Miltenyi Biotec). Cells were acquired on a FACSAria flow cytometer (BD Biosciences) and analyzed using Cyflogic software.

### Apoptosis

The percentage of lymphocytes undergoing apoptosis was determined by flow cytometry using AnV (annexin V)-FITC (Biolegend). The staining was performed according to the manufacturer's instructions.

### MDSC purification

Positive selection of Gr1^+^ cells was performed using microbeads and MACS LS columns (Miltenyi Biotec), according to the manufacturer's protocol. Positively selected cells were passed over a second selection column to increase their purity. Purity of selected cells was >95% as verified by flow cytometry. Cell viability was ≥95% as determined by Trypan blue exclusion.

### *In vitro* proliferation assay

Splenocytes were labeled with carboxyfluorescein diacetate succinimidyl ester (CFSE) according to the manufacturer's instructions (Biolegend). Following labeling, splenocytes were cultured in RPMI medium supplemented with 2 mM glutamine, 50 μM 2-βME, 50 μg/ml gentamicin and 10% FBS in triplicate in round-bottomed 96-well plates at 2 × 10^5^ cell/well (100 μl/well). Cells were cultured with 100 μl of complete medium or 100 μl of complete medium containing concanavalin A (5 μg/ml) for 72 h at 37°C and 5% CO_2_. Cells were then harvested and the supernatant was reserved to −20°C for cytokine quantification. Cells were stained with phycoerythrin-labeled anti-CD4 (Ligatis), allophycocyanin-labeled anti-CD8 (Biolegend). The percentage of CD4^+^ T cells and CD8^+^ T cells labeled with CFSE was determined by flow cytometry.

### *In vitro* suppression assay

Splenocytes from naïve mice were labeled with CFSE according to the manufacturer's instructions (Biolegend). Following labeling, splenocytes were cultured with purified MDCS obtained from the spleen of *S. aureus* inoculated mice in 1:0, 1:10, 1:20 (T cells:MDSC) ratio in RPMI medium supplemented with 2 mM glutamine, 50 μM 2-βME, 50 μg/ml gentamicin and 10% FBS in round-bottomed 96-well plates. Cells were cultured with complete medium containing concanavalin A (5 μg/ml) for 72 h at 37°C and 5% CO_2_. Cells were then harvested and the supernatant was reserved to −20°C for cytokine quantification. Cells were stained with phycoerythrin-labeled anti-CD4 (Ligatis), allophycocyanin-labeled anti-CD8 (Biolegend). The percentage of CD4^+^ T cells and CD8^+^ T cells labeled with CFSE was determined by flow cytometry.

### Real-time polymerase chain reaction

RNA was isolated using TRIzol Reagent (Invitrogen). Complementary DNA (cDNA) was made from 1 μg of RNA using M-MLV Reverse Transcriptase (Promega). The following primers were used for amplification: for mouse Arginase, 5′-GTC CCT AAT GAC AGC TCC TTT C-3′ and 5′-CCA CAC TGA CTC TTC CAT TCT T-3′; for mouse iNOS, 5′-CAC AGC AAT ATA GGC TCA TCC A-3′ and 5′-GGA TTT CAG CCT CAT GGT AAA C-3′; for mouse S100A8, 5′-GGA AAT CAC CAT GCC CTC TAC AA-3′ and 5′-ATG CCA CAC CCA CTT TTA TCA CC-3′; for mouse S100A9, 5′-GGA GCG CAG CAT AAC CAC CAT C-3′ and 5′-GCC ATC AGC ATC ATA CAC TCC TCA-3′ and for mouse glyceraldehyde-3-7phosphate dehydrogenase (GAPDH), 5′-AAC TTT GGC ATT GTG GAA GGG CTC-3′ and 5′-ACC CTG TTG CTG TAG CCG TAT TCA-3′. GAPDH was used as a control for standardization.

### ELISA

IL-10, IL-6, and IFN-γ were quantified by enzyme-linked immunosorbent assay using matched antibody pairs (BD Biosciences) according to the manufacturer's instructions.

### Statistical analysis

When comparing two groups of data Student's *t*-test or the nonparametric Mann Whitney *U*-test were used based on the data distribution. The proportions were analyzed using Fisher's exact test. The nonparametric correlation Spearman test was used to analyze the correlation. All the data collected from the experiments were included in the analysis. A *p*-value < 0.05 was considered statistically significant. GraphPad Prism software was used for statistical analysis.

## Results

### T cell anergy induced by *S. aureus* is dependent on TNFR1 signaling

**Figure 1:**
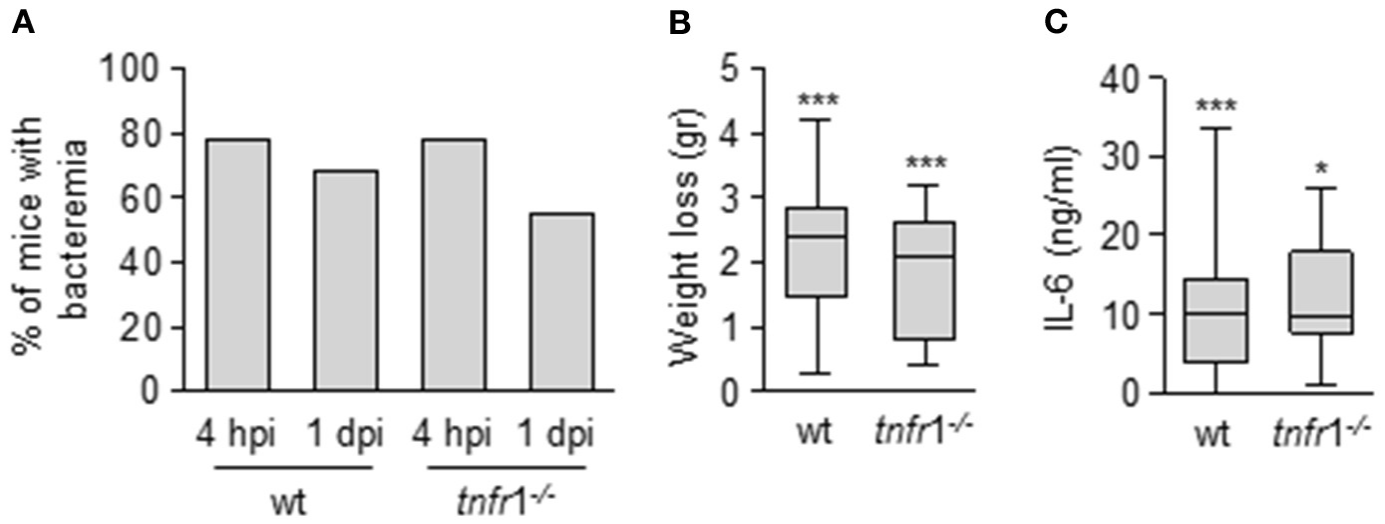
Impact of TNFR1 signaling in the early events after the onset of *S. aureus* sepsis of peritoneal origin. Groups of C57BL/6 wild type and *tnfr*1^−/−^ mice were inoculated with *S. aureus* FPR3757 by intraperitoneal route. **(A)** The presence of bacteria in blood were monitored 4 h and 1 day post-inoculation. Bars represent the percentage of mice with bacteriemia. **(B)** Weight loss were quantified at day 1 post-inoculation. **(C)** Plasma levels of IL-6 4 h post-inoculation were quantified by ELISA. The levels of IL-6 before inoculation were below the detection limit (15 pg/ml). **(B,C)** Boxes and whiskers depict maximum and minimum values obtained from individual mice and the horizontal line represents the median for each group. *p < 0.05, ***p < 0.001, compared with values obtained from the same group before inoculation, Mann-Whitney *U*-test for nonparametric data. hpi: hours post-inoculation, dpi: days post-inoculation.

In order to understand the role of TNFR1 signaling in the events that govern the immune dysfunction during *S. aureus* sepsis we determined the proliferative capacity of splenic T cells in the presence or absence of this receptor. The experiments were conducted using a mouse model of *S. aureus* sepsis of peritoneal origin (Ahn et al., 2006; Giai et al., 2013). In this model the onset of sepsis was not dependent on TNFR1 signaling (Figure 1) which allowed to determine the role of this receptor at a later stage. Infection was characterized by a high rate of bacteriemia 4 h after the inoculation of *S. aureus* (Figure 1A). Bacteria were present in blood 24 h later (Figure 1A) and cleared by day 4 (data not shown). Significant weight loss was also observed at day 1 (Figure 1B) and a 10% of mortality that occurred during the first 48 h was recorded in both the wild type and the *tnfr*1^−/−^ group. In addition, significantly increased levels of IL-6, which have been associated to the severity of sepsis in humans and animal models (Hack et al., 1989; Borrelli et al., 1996; Remick et al., 2002), were found in serum 4 h post-inoculation (Figure 1C).

**Figure 2:**
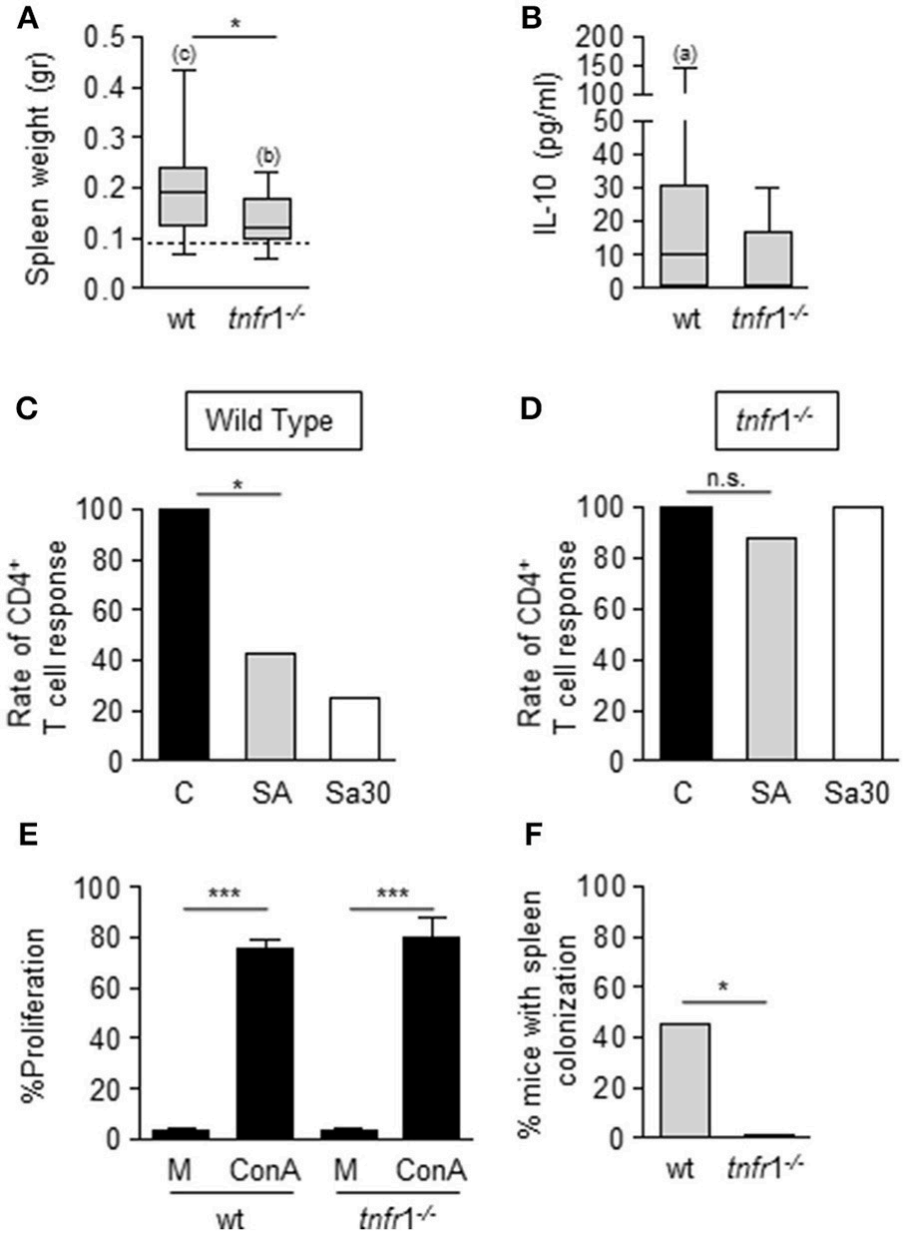
Impact of TNFR1 signaling on the local and systemic host response during *S. aureus* sepsis of peritoneal origin. **(A–F)** Groups of C57BL/6 wild type and *tnfr*1^−/−^ mice were inoculated with *S. aureus* FPR3757 (SA, gray bars and boxes), *S. aureus* Sa30 (Sa30, white bars) or PBS (C, black bars) by intraperitoneal route. **(A,B)** Spleen weight **(A)** and plasma levels of IL-10 **(B)** were determined at day 8 post-inoculation. IL-10 was not detected in plasma from mice inoculated with PBS. Boxes and whiskers depict maximum and minimum values obtained from individual mice and the horizontal line represents the median for each group. Dotted line in **(A)** depicts median value of the PBS group (wild type and *tnfr*1^−/−^ combined) *p < 0.05, (a) *p* < 0.05, (b) *p* < 0.01, (c) *p* < 0.001, (a, b, c) compared with the corresponding groups inoculated with PBS, Mann-Whitney *U*-test for nonparametric data. **(C,D)** Bars represent the percentage of mice with CD4^+^ T cells that responded to ConA stimulation (less than 10% decrease in the proliferative capacity compared with the control group). *p < 0.05, Fisher's exact test. **(E)** Splenocytes from mice inoculated with PBS were stimulated with medium or ConA (5 μg/ml). Bars represent the proliferative capacity of CD4^+^ T cells in response to medium or ConA stimulation. ***p < 0.001, Unpaired *t-*test. **(F)** Bars represent the percentage of mice with spleen colonization at day 8 post-inoculation. *p < 0.05, Fisher's exact test.

At day 8 after inoculation, surviving mice developed splenomegaly with a significant increase in the spleen mass (Figure 2A) and increased levels of IL-10 in serum, characteristic of the immunosuppressive phase of sepsis, were observed (Figure 2B). Interestingly, the splenomegaly was significantly lower in the absence of TNFR1 signaling (Figure 2A) and the levels of circulating IL-10 were also reduced in this group compared with wild type mice (Figure 2B). At this time point, a significant proportion of wild type septic mice showed decreased proliferative rates of CD4^+^ T cells (Figure 2C) whereas the same population from septic *tnfr*1^−/−^ mice was responsive to Con A (Figure 2D). The impact of TNFR1 signaling on T cell anergy did not depend on the staphylococcal strain used as similar results were obtained using the Sa30 clinical isolate for the induction of sepsis (Figures 2C,D). Moreover, we ruled out potential differences in proliferation inherent to the TNFR1 signaling by comparing the ability of CD4^+^ T cells from wild type and *tnfr*1^−/−^ mice inoculated with PBS to proliferate in response to ConA (Figure 2E). The increased functionality of CD4^+^ T cells from TNFR1 deficient mice correlated with the increased capacity of this group to clear bacteria from the spleen (Figure 2F). Taken together these results indicate that during staphylococcal sepsis CD4^+^ T cell anergy is significantly dependent on TNFR1 signaling and it may have an impact on bacterial burden in the spleen.

### MDSC modulate T cell activity during staphylococcal sepsis

**Figure 3:**
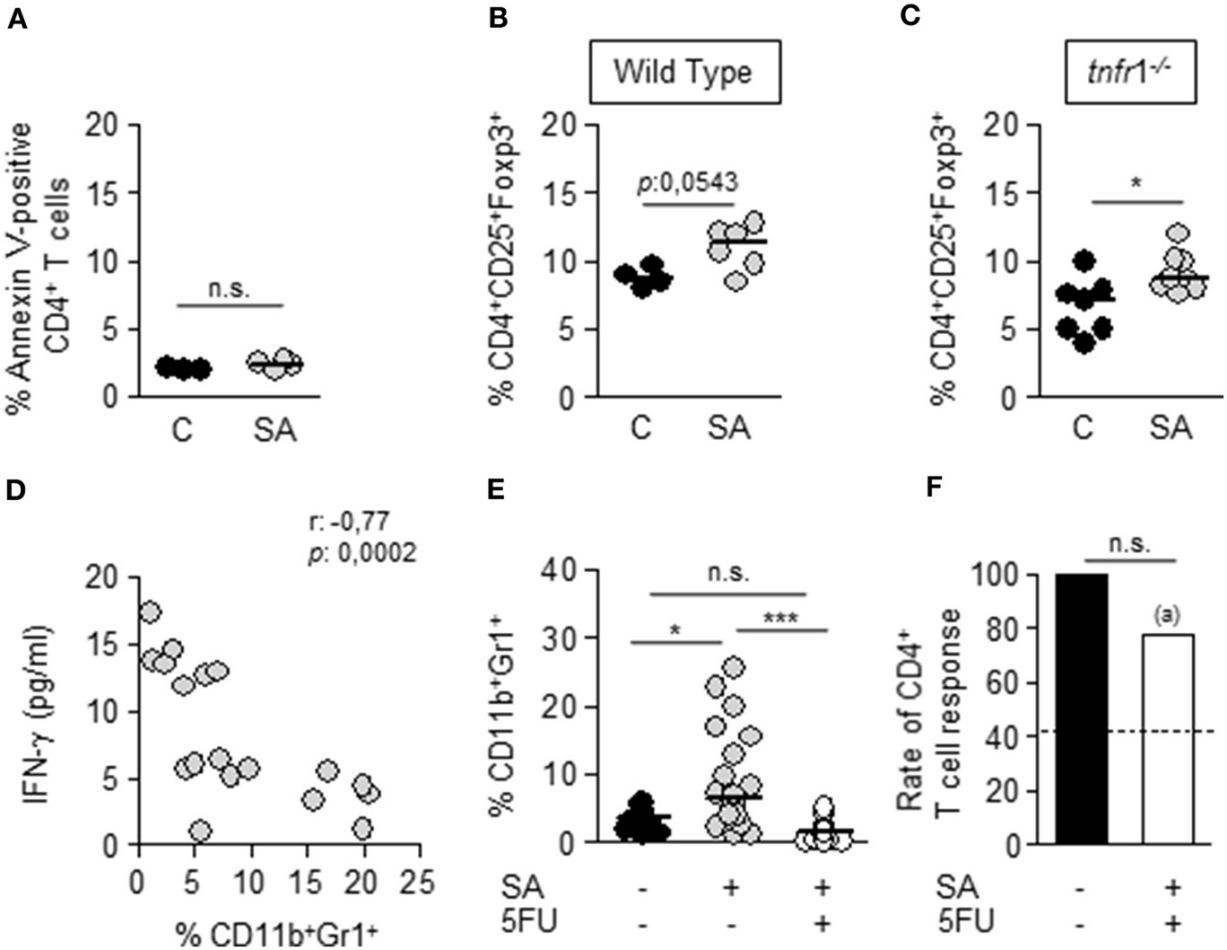
Mechanisms involved in T cell dysfunction during *S. aureus* sepsis. Groups of C57BL/6 wild type **(A,B, D–F)** and *tnfr*1^−/−^ **(C)** mice were inoculated with *S. aureus* FPR3757 (SA, gray circles) or PBS (C, black circles and bars) by intraperitoneal route and treated or not with 5FU (50 mg/Kg) (white bar) at day 4 post-inoculation by the same route. **(A–C)** The percentage of Annexin V-positive CD4^+^ T cells **(A)** and the percentage of Treg cells (CD4^+^CD25^+^Foxp3^+^) **(B,C)** present in the spleen at day 8 post-inoculation were quantified. Each circle represents an individual mouse and the horizontal lines indicate the median value for each group. *p < 0.05, Mann-Whitney *U*-test for nonparametric data. **(D)** Correlation analysis between the levels of IFN-γ produced by splenocytes of *S. aureus* inoculated mice in response to ConA and the percentage of CD11b^+^Gr1^+^ cells present in the spleen of each mice at the time of splenocyte isolation. Each circle represent an individual mouse. Data were analyzed using the Spearman test. **(E)** Percentage of MDSC present in the spleen at day 8 post-inoculation. *p < 0.05, ***p < 0.001, Mann-Whitney *U*-test for nonparametric data. **(F)** Bars represent the percentage of mice whose CD4^+^ T cells were able to respond to ConA stimulation (less than 10% decrease in the proliferative capacity compared with the control group). Dotted line indicates the rate of CD4^+^ T cell response in mice inoculated with SA and not treated with 5FU. (a) *p* < 0.05, compared with *S. aureus* inoculated mice not treated with 5FU; n.s. not significant, Fisher's exact test.

Several mechanisms have been proposed to account for T cell dysfunction during sepsis. Among them are apoptosis of T cells (Hotchkiss et al., 1999, 2001, 2002; Muenzer et al., 2010), expansion of Treg (CD25^+^CD4^+^Foxp3^+^) cells (Hotchkiss et al., 2013; Stieglitz et al., 2015) as well as the accumulation of MDSC in peripheral organs (Sander et al., 2010; Cuenca et al., 2011). The viability of CD4^+^ T cells in the spleen of infected wild type mice was equivalent to that of the control group (Figure 3A) suggesting that apoptosis of T cells is not a major process at this time point during staphylococcal infection. A small increase in the percentage of Tregs was observed in both wild type and *tnfr*1^−/−^ mice at day 8 after inoculation (Figures 3B,C) suggesting a minor role of this population in the TNFR1-dependent T cell anergy. Then, we determined the contribution of MDSC to the *S. aureus*-induced T cell anergy. The levels of IFN-γ produced by splenocytes from wild type infected mice in response to Con A, although they were very variable among animals, showed significant inverse correlation with the percentage of MDSC present in the spleen of wild type septic mice (Figure 3D). To further confirm the role of MDSC on the T cell dysfunction, the expansion of this population was pharmacologically inhibited. Groups of wild type mice were challenged with *S. aureus* and 5-Fluorouracil (5FU), and inducer of MDSC death (Vincent et al., 2010), was administered by intraperitoneal route 4 days later, a time at which bacteria had already colonized the spleen and MDSC started to accumulate in this organ (data not shown). As expected, the percentage and the number of CD11b^+^Gr1^+^ cells in the spleen of mice treated with 5-FU was significantly lower than that found in *S. aureus* infected, non-treated mice and comparable to control non-infected mice (Figure 3E and data not shown). In the absence of MDSC accumulation the capacity of CD4^+^ T cells to proliferate in response to Con A was restored (Figure 3F) indicating a major role for MDSC in the generation of anergic CD4^+^ T cells during *S. aureus* sepsis.

### Accumulation of MDSC in the spleen is not dependent on TNFR1 signaling

**Figure 4:**
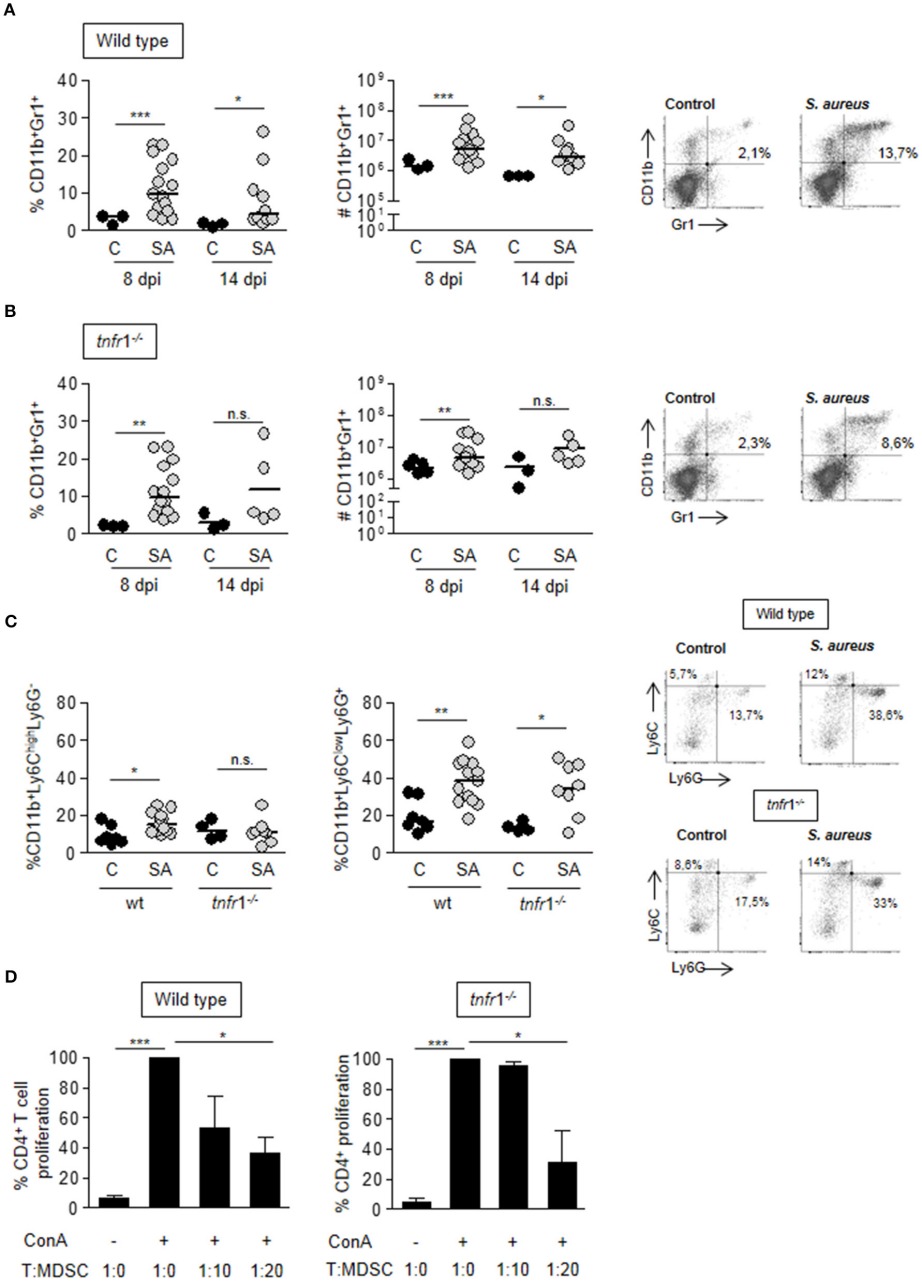
MDSC expansion and accumulation in the spleen during *S. aureus* sepsis. **(A–D)** Groups of C57BL/6 wild type **(A,C)** and *tnfr*1^−/−^ **(B,C)** mice were inoculated with *S. aureus* FPR3757 (SA, gray circles) or PBS (C, black circles) by intraperitoneal route. **(A,B)** The percentage and the absolute number of CD11b^+^Gr1^+^ cells present in the spleen was determined at days 8 and 14 post-inoculation by staining with specific antibodies and flow cytometry analysis. Representative dot plots and percentages of cells gated as CD11b^+^Gr1^+^ at day 8 post-inoculation are shown. **(C)** CD11b, Ly6G, and Ly6C expression was determined in splenocytes at day 8 post-inoculation. Results were calculated after gating on the CD11b^+^ population. Representative dot plots of Ly6G and Ly6C-stained cells and the quantitation of Mo-MDSC: Ly6C^high^Ly6G^−^ and PMN-MDSC: Ly6C^low^Ly6G^+^ are shown. *p < 0.05, **p < 0.01, ***p < 0.001, Mann-Whitney *U*-test for nonparametric data. The quadrants were placed according to the isotype controls. **(D)** Splenocytes from naive mice were co-cultured with different proportions of CD11b^+^Gr1^+^ cells purified from *S. aureus* inoculated mice at day 8 post-challenge and stimulated with ConA (5 μg/ml). Bars represent proliferation of CD4^+^ T cells relative to that observed in the absence of MDSC (considered as 100%). *p < 0.05, ***p < 0.001, Unpaired *t-*test.

After demonstrating that both TNFR1 signaling and MDSC are critical in the induction of T cell anergy during staphylococcal sepsis we evaluated the putative relationship between the signaling mediated by this receptor and MDSC expansion and accumulation in the spleen. A significant accumulation of CD11b^+^Gr1^+^ cells in the spleen was observed at day 8 after the onset of sepsis in both wild type and *tnfr*1^−/−^ mice (Figures 4A,B). The absolute number of splenic CD11b^+^Gr1^+^ cells in septic mice was 6-fold the corresponding one in control mice and increased levels of this population were still present in the spleen at day 14 post-inoculation (Figure 4A). Septic *tnfr*1^−/−^ mice exhibited a similar pattern of MDSC accumulation in the spleen (Figure 4B) indicating that this process is independent of TNFR1 signaling. The relative proportions of cells with monocytic and granulocytic morphology among this immature population was determined using the Ly6G and Ly6C surface markers. Granulocytic MDSC were the prevalent phenotype during infection in both wild type and *tnfr*1^−/−^ mice (Figure 4C). The suppressor phenotype of the MDSC expanded and accumulated in the spleen in wild type and *tnfr*1^−/−^ mice during *S. aureus* infection was confirmed by determining their capacity to suppress the proliferation of naïve T cells in response to Con A *in vitro* (Figure 4D).

**Figure 5:**
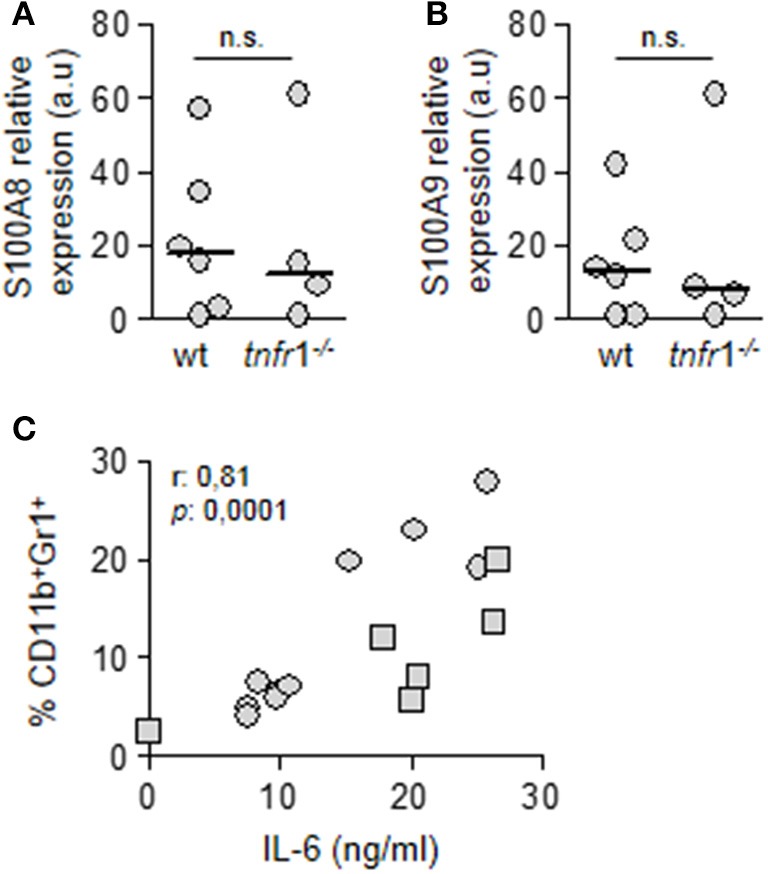
Role of TNFR1 in the accumulation of MDSC in the spleen. Groups of C57BL/6 wild type and *tnfr*1^−/−^ mice were inoculated with *S. aureus* FPR3757 by intraperitoneal route. **(A,B)** MDSC were purified from the spleen at day 8 post-inoculation. The relative expression levels of S100A8 **(A)** and S100A9 **(B)** mRNA in this population were determined by RT real-time PCR and normalized to GAPDH. (a.u.): arbitrary units. Mann-Whitney *U*-test for nonparametric data. **(C)** Correlation analysis between plasmatic levels of IL-6 in C57BL/6 wild type (squares) and *tnfr*1^−/−^ (circles) mice 4 h post-inoculation and the percentage of MDSC present in the spleen at day 8 post-inoculation. Data were analyzed using the Spearman test.

Purified MDSC from both wild type and *tnfr*1^−/−^ mice expressed similar levels of S100A8 and S100A9 (Figures 5A,B) which have a known role in inflammation and directing recruitment of this population (Cheng et al., 2008; Sinha et al., 2008). This is in line with our results that indicate that MDSC recruitment is not dependent of TNFR1 signaling. Among the cytokines that have been proposed to induce MDSC accumulation in tumors is IL-6 (Mundy-Bosse et al., 2011; Tsukamoto et al., 2013; Chen et al., 2014). In the sepsis model, IL-6 is early induced during infection (Figure 1C) (Giai et al., 2016) and a significant correlation between the percentage of splenic MDSC and the levels of circulating IL-6 at the onset of sepsis was observed in both groups of mice (Figure 5C) suggesting that it might be implicated in MDSC accumulation in the spleen.

### TNFR1 signaling has an impact in the expression of immunomodulatory mediators by MDSC

**Figure 6:**
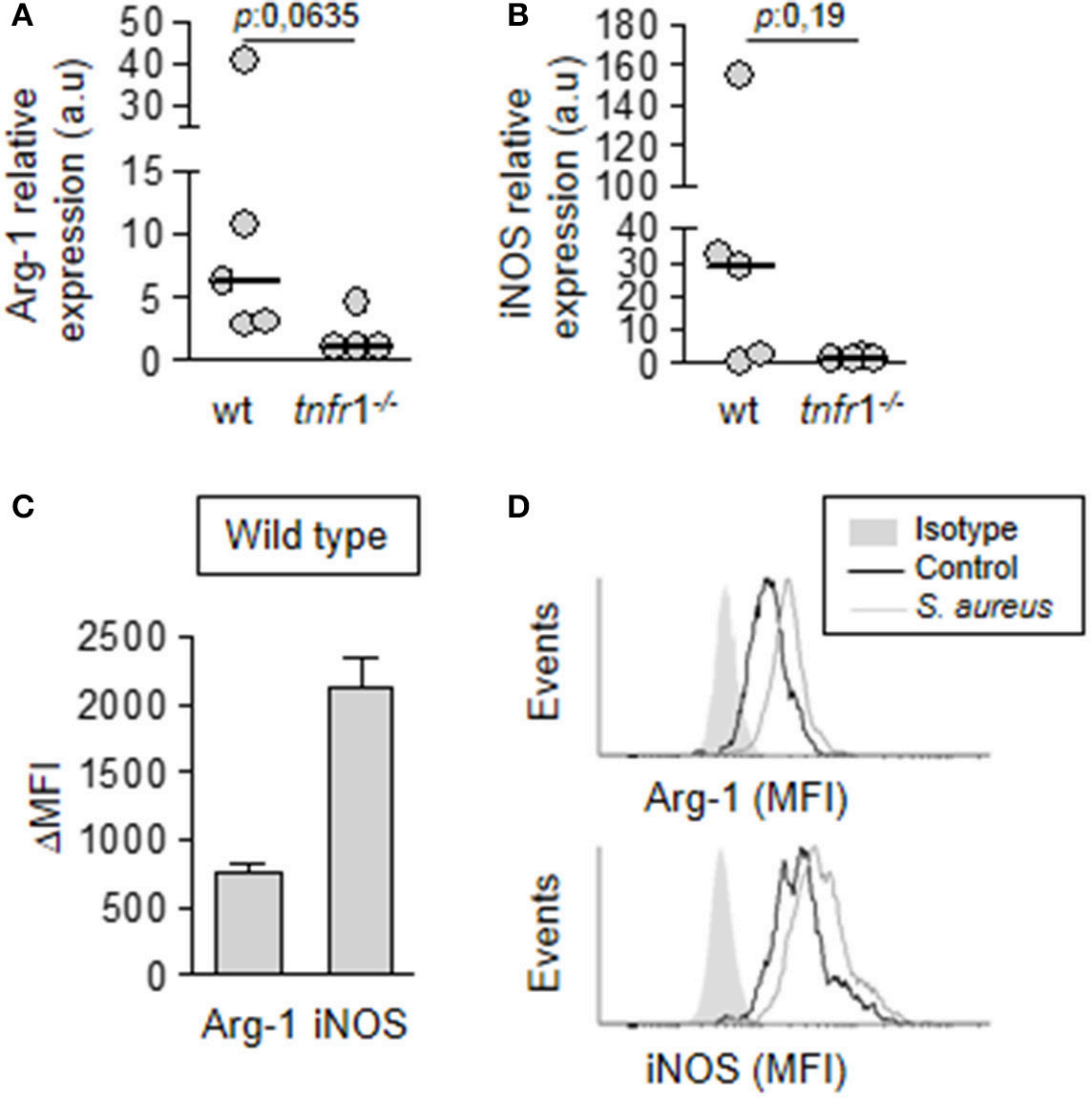
Expression of immunosuppressive mediators by MDSC. Groups of C57BL/6 wild type **(A–D)** and *tnfr*1^−/−^ **(A,B)** mice were inoculated with *S. aureus* FPR3757 (SA, gray circles and bars) by intraperitoneal route. **(A,B)** The MDSC present in the spleen were purified at day 8 post-inoculation and the relative expression of Arg-1 **(A)** and iNOS **(B)** in this population was determined by RT real-time PCR and normalized to GAPDH. (a.u.): arbitrary units. Mann-Whitney *U*-test for nonparametric data. **(C,D)** The presence of Arginase and iNOS was determined by intracellular staining and flow cytometry in MDSC from wild type mice inoculated with *S. aureus* or PBS (control). **(C)** ΔMFI: Mean Fluorescence Intensity (MFI) infected - MFI control. **(D)** Representative histograms are shown.

After demonstrating that the T cell anergy observed during staphylococcal sepsis was dependent on both, TNFR1 signaling and MDSC, but the accumulation of MDSC in the spleen of *tnfr*1^−/−^ was equivalent to that of wild type mice we hypothesized that TNFR1 signaling could modulate the expression of immunosuppressive mediators in MDSC. These cells are known to exert their suppressive function through the expression of Arginase 1 and iNOS (Trikha and Carson, 2014; Park et al., 2018). Therefore, we evaluated the expression of these enzymes in MDSC purified from the spleen of wild type and *tnfr*1^−/−^ mice at day 8 after the onset of sepsis. MDSC from wild type septic mice expressed both Arg-1 (Figure 6A) and iNOS (Figure 6B). Conversely, expression levels of these enzymes were low or undetectable in MDSC from *tnfr*1^−/−^ mice (Figures 6A,B). The expression of Arginase 1 and iNOS in wild type mice at the protein level was confirmed by intracellular staining and flow cytometry (Figures 6C,D). These results indicate that whereas TNFR1 signaling is not required for MDSC accumulation and expansion in the spleen, it determines the *in vivo* expression of enzymes known to participate in the suppressive function of this population.

**Figure 7:**
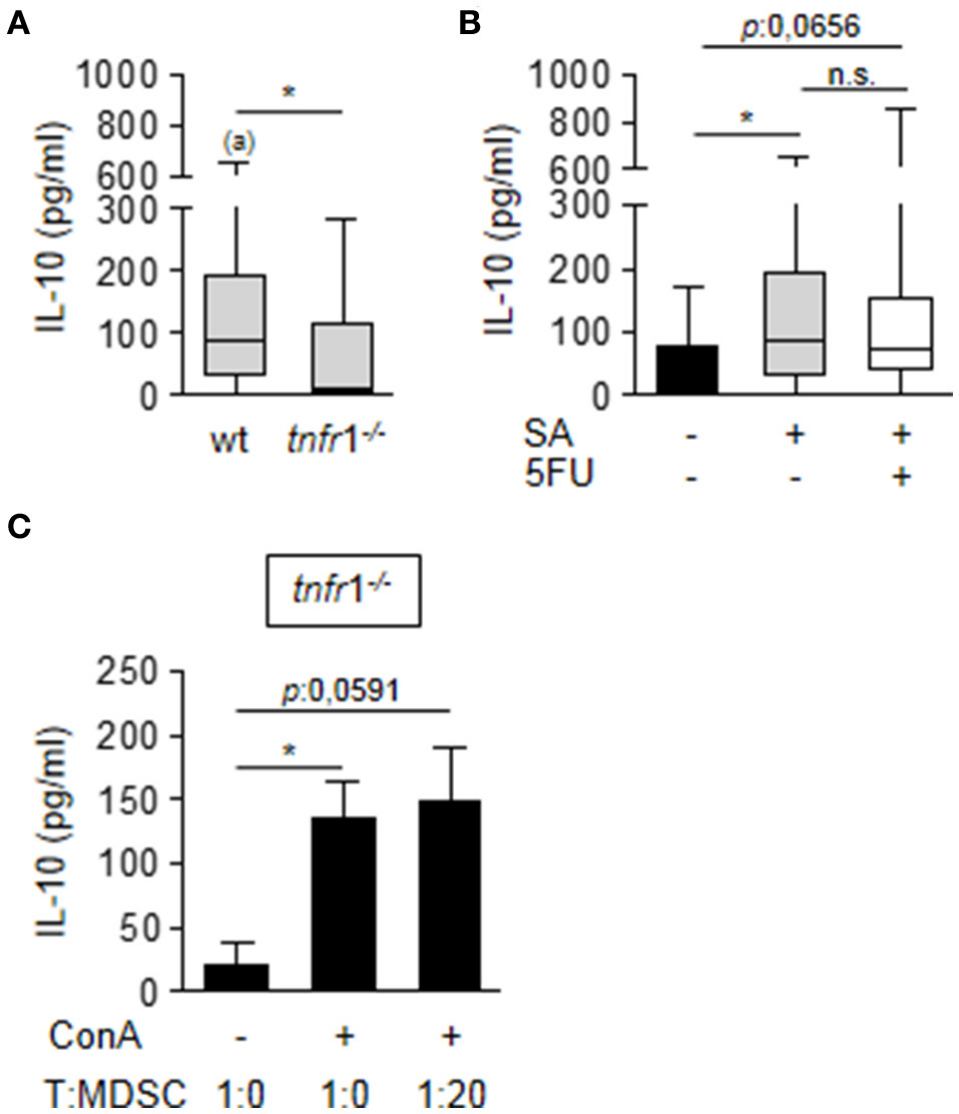
Impact of TNFR1 expression in the production of IL-10. **(A,B)** Groups of C57BL/6 wild type **(A,B)** and *tnfr*1^−/−^ **(A)** mice were inoculated with *S. aureus* FPR3757 (SA, gray boxes and bars) by intraperitoneal route and treated or not with 5FU (50 mg/Kg) (white box) at day 4 post-inoculation. IL-10 production in the spleen was quantified by ELISA. Boxes and whiskers depict maximum and minimum values obtained from individual mice and the horizontal line represents the median for each group. *p < 0.05, (a) *p* < 0.05 (compared with wild type mice inoculated with PBS) Mann-Whitney *U*-test for nonparametric data. **(C)** Splenocytes from naïve *tnfr*1^−/−^ mice were co-cultured with different proportions of CD11b^+^Gr1^+^ cells purified from *tnfr*1^−/−^ mice inoculated with *S. aureus* and stimulated with ConA (5 μg/ml). Bars represent IL-10 levels in the supernatant at 72 h post-stimulation. *p < 0.05, Mann-Whitney *U*-test for nonparametric data.

Considering that MDSC from TNFR1 deficient mice showed suppressive activity after *in vitro* culture in the presence of ConA (Figure 4D), it is suggested that environmental factors in the spleen that are modulated by TNFR1 could be involved in the regulation of Arginase 1 and iNOS expression by MDSC *in vivo*. Among the factors that could modulate MDSC activity is IL-10 (Pinton et al., 2016; Chen et al., 2017). Therefore, we determined the induction of IL-10 in the spleen of wild type and TNFR1 deficient septic mice. *S. aureus* infection induced IL-10 in both groups (Figure 7A). However, wild type mice presented significantly increased levels of this cytokine in the spleen compared with *tnfr*1^−/−^ mice (Figure 7A). Treatment with 5FU did not impact the overall production of IL-10 (Figure 7B) and the production of this cytokine was not detected in purified MDSC from wild type mice (data not shown) suggesting that although TNFR1 signaling was involved in the induction of this cytokine in response to *S. aureus*, MDSC are not the primary source of IL-10 in the spleen during staphylococcal sepsis. To further investigate the role of IL-10 in the regulation of MDSC activity we quantified the levels of this cytokine in the supernatant of the *in vitro* co-cultures of MDSC from infected *tnfr*1^−/−^ mice and näive splenocytes stimulated with ConA as it was shown in Figure 4D. Stimulation of splenocytes with ConA induced the production of IL-10 even in the absence of TNFR1 signaling (Figure 7C) which could explain the suppressive capacity of MDSC during the *in vitro* co-culture experiments. Therefore, these results suggest that TNFR1-mediated IL-10 production in response to *S. aureus* infection may modulate MDSC function during staphylococcal sepsis rather than TNFR1 direct signaling on MDSC.

## Discussion

*S. aureus* has emerged as a leading cause of sepsis (Powers and Bubeck Wardenburg, 2014). Through improvements in supportive care measures over the last few decades an increasing percentage of the patients survive through the initial systemic inflammatory period but a significant proportion of the patients eventually succumb to late nosocomial infections (Cuenca and Moldawer, 2012). The high mortality rate of sepsis and the limited clinical success of multiple clinical trials aimed at modulating the host immune response (Russell, 2006) indicate that there is very limited understanding of the complex host-pathogen interactions that take place during this pathology. Although there is a growing consensus that late sepsis-associated morbidities are related to global immunosuppression, the multiple factors that drive the suppressive immune response are not completely elucidated.

In the present study we demonstrated that TNFR1 signaling is critically involved in the development of T cell anergy during staphylococcal sepsis. To assure that this finding was not unique to the infecting bacterial strain, we used two unrelated isolates, the MRSA USA300 FPR3757 designated as sequence type ST8 and highly epidemic in the United States (Planet, 2017) and the MRSA Sa30 designated as sequence type ST30, the most prevalent clone isolated from invasive community acquired staphylococcal infections in Argentina (Fernandez et al., 2013). TNF-α has been previously linked to CD4^+^ T cell anergy in HIV infection (Kaneko et al., 1997) and TRAFs, signaling molecules downstream of TNFR1, have also been implicated in defective T cell function (So et al., 2015). During staphylococcal sepsis, CD4^+^ T cell anergy does not seem to be the consequence of direct TNFR1 signaling on T cells because their functionality could be recovered by blocking the expansion of MDSC.

MDSC have been previously linked to T cell anergy and bacterial persistence during chronic *S. aureus* infection (Heim et al., 2014; Tebartz et al., 2015). Moreover, in recent studies conducted with septic patients the expansion of granulocytic MDSC correlated with bad prognosis of the disease regardless of the microorganism involved (Mathias et al., 2017). Our results further confirm that MDSC are major modulators of T cell activity during staphylococcal sepsis. Moreover, T cell anergy induced by *S. aureus* was accompanied by a heterogeneous production of IFN-γ in the spleen and the levels of this cytokine negatively correlated with the percentage of splenic MDSC. This observation might be of clinical interest because it has been shown that administration of IFN-γ reversed the adverse sequelae of sepsis induced immunosuppression in patients (Döcke et al., 1997).

Despite the increasing recognition of MDSC as key players in modulating suppressive responses during infection there is very little knowledge of the mechanisms involved in their recruitment, differentiation arrest and acquisition of the immunosuppressive phenotype. TNF-α signaling mediates many of the systemic inflammatory consequences of sepsis (Chousterman et al., 2017). It is also critical for *S. aureus* clearance (Nakane et al., 1995; Skabytska et al., 2014). Our results in the *S. aureus* sepsis model, demonstrated that TNF-α signaling through TNFR1 is not required for MDSC expansion and accumulation but it is critical for the expression of MDSC immunosuppressive mediators such as Arginase 1 and iNOS. A two signal model has been proposed for the expansion and function of MDSC in which a first signal is responsible for MDSC expansion and the second one for driving MDSC activation (Condamine and Gabrilovich, 2011). During chronic inflammation induced by mycobacterial antigens, TNF-α exhibited a dual function arresting differentiation of immature myeloid cells and augmenting MDSC activity (Sade-Feldman et al., 2013). In tumors, it was proposed that TNF-α signaling through TNFR2 inhibits caspase 8 activity and promotes MDSC survival and accumulation helping tumor cells to evade the immune system (Zhao et al., 2012). More recently the role of TNFR2 in the generation of monocytic MDSC has been demonstrated (Polz et al., 2014). TNFR2 signaling is mainly driven by membrane associated TNF-α whereas TNFR1 signaling is triggered by soluble TNF-α (Cabal-Hierro and Lazo, 2012). We have previously demonstrated that *S. aureus* activates ADAM17, the metalloprotease responsible of cleaving TNF-α from the cell membrane. Moreover, in the sepsis model TNF-α expression is induced in response to *S. aureus* inoculation (Giai et al., 2013). Therefore, it is not unexpected that TNFR1 signaling will be active during staphylococcal sepsis.

Interestingly, MDSC isolated from *tnfr*1^−/−^ septic mice showed intrinsic suppressive capacity when evaluated *in vitro*, which suggests that mediators locally produced during infection in a TNFR1-dependent manner could modulate their activity *in vivo*. In line with this hypothesis, recent findings show that locally produced IL-10 is implicated in MDSC activation in an *S. aureus* biofilm infection model (Heim et al., 2015) and in a collagen-induced model of rheumatoid arthritis (Park et al., 2018). The data of our work indicate that during *S. aureus* sepsis, TNFR1 signaling was critical for systemic and local IL-10 induction. This finding could explain the lower expression of Arginase 1 and iNOS, both known immunosuppressive factors, in MDSC from *tnfr*1^−/−^ mice compared with MDSC from wild type mice during *in vivo* infection. Therefore, the microenvironment induced in response to the signaling cascades initiated by TNFR1, which among other putative factors contains IL-10, could provide the “second signal” and determine the activity of MDSC during staphylococcal sepsis. In this regard, it has been established that IL-10 levels are increased in septic patients and that those levels predict mortality (Hotchkiss and Karl, 2003). The source of IL-10 in the spleen were not the MDSC as this cytokine was not detected in isolated MDSC and the levels of IL-10 were not affected by blocking the expansion of this population. Thus, it remains to be elucidated the population responsible for TNFR1-dependent induction of IL-10.

Regarding the nature of the “first signal” driving the expansion and accumulation of MDSC several studies have demonstrated a positive relationship between increased proportions of MDSC and higher levels of IL-6 (Trikha and Carson, 2014; Chen et al., 2017). Our results show a positive correlation between the levels of IL-6 at early times after inoculation and the percentage of MDSC accumulated in the spleen similarly to what it is observed in septic patients in which a positive correlation between MDSC and the levels of IL-6 at the day of admission was found (Darcy et al., 2014).

In previous attempts to understand the events that dictate the immunosuppression during sepsis, a role for Treg cells has been proposed (Hotchkiss et al., 2013; Stieglitz et al., 2015). Moreover, the capacity of MDSC in inducing the expansion of Tregs has been demonstrated (Park et al., 2018). According to our study, the Treg population seems to have, if any, a minor role in the suppression of CD4^+^ T cell responses during staphylococcal sepsis based on the observation that the expansion of these cells did not correlate with either proper o defective TNFR1 signaling.

T cell anergy has now been largely recognized as an important component of sepsis-related immunosuppression. However, the signaling pathways induced by different bacterial pathogens that lead to dampened T cell function are poorly understood. TNFR1 is a central player during *S. aureus* infections. This receptor not only signals TNF-α, which is rapidly induced after the entry of the bacteria, but it also recognizes protein A, a major staphylococcal surface protein, initiating both pro-inflammatory (Gómez et al., 2004, 2006) and anti-inflammatory cascades (Giai et al., 2013, 2016). The results presented here indicate that during staphylococcal sepsis TNFR1 has a significant impact in modulating CD4^+^ T cell anergy and that function of this receptor is relevant to bacterial clearance in the spleen. Moreover, we demonstrated that TNFR1 signaling induces the microenvironment to sustain MDSC suppressive activity demonstrating the importance of this receptor during the immunosuppressive phase of staphylococcal sepsis. Whether TNFR1 signaling triggered by protein A, in addition to TNF-α, is critical for MDSC activity remains to be elucidated.

## Author contributions

MG and CL conceived and designed the experiments, analyzed the data and wrote the manuscript. CL, CG, and CP performed the experiments. CG, CP, and MM analyzed the data and critically revised the manuscript. MG procured funding. All authors read and approved the final manuscript.

### Conflict of interest statement

The authors declare that the research was conducted in the absence of any commercial or financial relationships that could be construed as a potential conflict of interest.
